# Association between Vitamin D Genetic Risk Score and Cancer Risk in a Large Cohort of U.S. Women

**DOI:** 10.3390/nu10010055

**Published:** 2018-01-09

**Authors:** Paulette D. Chandler, Deirdre K. Tobias, Lu Wang, Stephanie A. Smith-Warner, Daniel I. Chasman, Lynda Rose, Edward L. Giovannucci, Julie E. Buring, Paul M. Ridker, Nancy R. Cook, JoAnn E. Manson, Howard D. Sesso

**Affiliations:** 1 Division of Preventive Medicine, Department of Medicine, Brigham and Women’s Hospital, Boston, MA 02115, USA; dtobias@partners.org (D.K.T.); lwang284@its.jnj.com (L.W.); Dchasman@partners.org (D.I.C.); lrose@rics.bwh.harvard.edu (L.R.); jburing@partners.org (J.E.B.); pridker@partners.org (P.M.R.); ncook@partners.org (N.R.C.); jmanson@partners.org (J.E.M.); hsesso@partners.org (H.D.S.); 2 Harvard Medical School, Boston, MA 02115, USA; swarner@hsph.harvard.edu (S.A.S.-W.); egiovann@hsph.harvard.edu (E.L.G.); 3 Department of Nutrition, Harvard T.H. Chan School of Public Health, Boston, MA 02115, USA; 4 Department of Epidemiology, Harvard T.H. Chan School of Public Health, Boston, MA 02115, USA; 5 Channing Division of Network Medicine, Department of Medicine, Brigham and Women’s Hospital, Boston, MA 02115, USA; 6 Cardiovascular Division, Brigham and Women’s Hospital, Boston, MA 02115, USA; 7 Mary Horrigan Connors Center for Women’s Health and Gender Biology, Brigham and Women’s Hospital, Boston, MA 02115, USA

**Keywords:** vitamin D, cancer, genetic risk score, Mendelian randomization, mortality

## Abstract

Some observational studies suggest an inverse association between circulating 25-hydroxyvitamin D (25OHD) and cancer incidence and mortality. We conducted a Mendelian randomization analysis of the relationship between a vitamin D genetic risk score (GRS, range 0–10), comprised of five single nucleotide polymorphisms (SNPs) of vitamin D status in the DHCR7, CYP2R1 and GC genes and cancer risk among women. Analysis was performed in the Women’s Genome Health Study (WGHS), including 23,294 women of European ancestry who were cancer-free at baseline and followed for 20 years for incident cancer. In a subgroup of 1782 WGHS participants with 25OHD measures at baseline, the GRS was associated with circulating 25OHD mean (SD) = 67.8 (26.1) nmol/L, 56.9 (18.7) nmol/L in the lowest versus 73.2 (27.9) nmol/L in the highest quintile of the GRS (*p* trend < 0.0001 across quintiles). However, in age-adjusted Cox proportional hazards models, higher GRS (reflecting higher 25OHD levels) was not associated (cases; Hazard Ratio (HR) (95% Confidence Interval (CI)), *p*-value) with incident total cancer: (*n* = 3985; 1.01 (1.00–1.03), *p* = 0.17), breast (*n* = 1560; 1.02 (0.99–1.05), *p* = 0.21), colorectal (*n* = 329; 1.06 (1.00–1.13), *p* = 0.07), lung (*n* = 330; 1.00 (0.94–1.06), *p* = 0.89) or total cancer death (*n* = 770; 1.00 (0.96–1.04), *p* = 0.90). Results were similar in fully-adjusted models. A GRS for higher circulating 25OHD was not associated with cancer incidence or mortality.

## 1. Introduction

Controversy remains whether chronic insufficiency of vitamin D is a causal determinant of incident cancer and mortality [1,2]. Although several observational studies indicate an inverse association between circulating 25-hydroxyvitamin D (25OHD) and cancer incidence and mortality, randomized trials assessing the effect of vitamin D supplementation on cancer incidence have not found clear benefits to date [2,3]. Mendelian randomization studies of single nucleotide polymorphisms (SNPs) influencing plasma level of 25OHD can be used to investigate the effect of lifelong differences in 25OHD and risk of cancer and mortality. If circulating 25-hydroxyvitamin D (25OHD) is causally related to cancer risk, then participants inheriting alleles predictive of low 25OHD concentrations may be at increased cancer risk compared to subjects with alleles predictive of high 25OHD.

Two recent genome-wide association studies (GWAS) [4,5] and other studies identified SNPs significantly associated with circulating 25OHD concentrations and vitamin D metabolism [6,7,8]. A recent publication reported that genetically low 25OHD concentrations were associated with increased all-cause mortality and cancer mortality in three large Danish cohorts [1]. The 25OHD SNPs used to calculate 25OHD genetically [1] included rs11234027 and rs12794714 in the DHCR7 gene encoding the enzyme that converts 7-dehydrocholesterol to cholesterol rather than vitamin D_3_ and rs10741657 and rs7944926 in the CYP2R1 gene, encoding the 25-hydroxylase that converts vitamin D to 25OHD in the liver [9]. Given the association of rs11234027, rs12794714, rs10741657 and rs794492 with genetically low 25OHD and increased all-cause mortality and cancer mortality, we evaluated these same four SNPs in addition to the rs2282679 in the GC gene encoding the vitamin D binding protein, the major transporter of circulating vitamin D compounds [9]. These five SNPs explain about 5% of the between-person variation in concentrations of circulating 25OHD [9]. We leveraged the Women’s Genome Health Study (WGHS) [10], a cohort of 23,294 women of European ancestry with genome-wide genotyped data, to derive a genetic risk score (GRS) of these SNPs. This instrumental variable was used to investigate the causal association of vitamin D deficiency and the risk of incident cancer and cancer mortality.

## 2. Methods

### 2.1. Women’s Genome Health Study 

The Women’s Health Study (WHS) began in 1992 and is a completed randomized, double-blind, placebo-controlled, 2 × 2 factorial trial that examined the role of aspirin (100 mg every other day) and vitamin E (600 IU every other day) in the primary prevention of cancer and cardiovascular disease (CVD) among 39,876 United States female health professionals aged 45 years and older. When the trial ended in 2004, 33,682 women (88.6% of those alive) consented to continue with observational follow-up, reporting on their health habits and medical history annually on questionnaires. The cohort continues to be monitored, now with greater than 20 years of follow-up. The WGHS is a majority subset of WHS women who agreed to participate in additional genomics analyses; baseline blood samples were used to extract DNA. The current analysis included 23,294 WGHS women of verified European ancestry free of cancer at baseline and who had provided baseline blood samples with available DNA. Of these, 1782 participants had both previously available 25OHD measurements and genotype data. 

All subjects provided written informed consent. The study was conducted in accordance with the Declaration of Helsinki, and the Institutional Review Board of Brigham and Women’s Hospital approved the protocol.

### 2.2. Cancer Endpoint Ascertainment

During follow-up, every 6 months in the first year and almost every year thereafter, participants received questionnaires that assessed their compliance, potential side effects, updated risk factors and outcomes of interest. When the trial ended in 2004, 33,682 women (88.6% of those alive) consented to continue with observational follow-up, reporting their health habits and medical history annually on questionnaires. Morbidity follow-up in the WHS was complete for 97.2% and mortality follow-up for 99.4% of women. For cases of cancer reported during the study period, subjects provided written consent for medical record review. A committee of physicians reviewed medical records. For the current analysis, we included confirmed invasive cancer cases through 2015. With a median follow-up of 20 years, 3985 confirmed cancer cases include 1560 breast, 329 colorectal, 330 lung cancers and 770 cancer deaths and 2973 total deaths. Endpoint review was complete for 95% of reported cancer cases. The confirmation rate among participants with records is 82%. Of all deaths, 60% have a cause confirmed by medical records and 85% are confirmed with death certificates or National Death Index reports.

### 2.3. Genotyping in the WGHS

Detailed methods of genotyping in the WGHS have been previously reported [10]. In brief, genotyping used the Illumina’s Infinium II assay [11] applied to the HumanHap300 Duo + platform (Illumina, San Diego, CA, USA). The final WGHS data included 23,294 participants of self-reported European ancestry confirmed by a multi-dimensional scaling procedure in PLINK10 (http://zzz.bwh.harvard.edu/plink/summary.shtml, PLINK10 is an open-source whole genome association analysis toolset). Genotyping was successful for rs11234027 and rs7944926 in 99.9% of the WGHS participants. The other SNPs, rs10741657, rs12794714, and rs2282679, were 1000G imputed (dosages of the 3 imputed SNPs were converted to genotypes by rounding to the closest genotype value).

### 2.4. Dietary and Lifestyle Factors

Body mass index (BMI), lifestyle (e.g., physical activity) and dietary data (e.g., vitamin D intake) were derived from the baseline questionnaire. The validity and reproducibility of a semi-quantitative food-frequency questionnaire (FFQ) have been described previously [12,13,14].

### 2.5. Laboratory Assessment of 25OHD

We combined samples from1782 women with previously-measured 25OHD in WGHS: a colorectal cancer case-control study [15], hypertension case/control subgroup, breast cancer case-control subgroup and a vitamin D pilot subgroup. Plasma 25OHD was measured at Heartland Assays, Inc. (Ames, IA, USA) with the FDA-approved direct, competitive chemiluminescence immunoassay (CLIA) using the DiaSorin LIAISON 25-OH Vitamin D Total assay (DiaSorin LIAISON 25OHD assay is a chemiluminsecent immunoassay for the quantitiative determination of 25OHD and other hydroxylated vitamin D metabolites). The assay utilizes a specific antibody to 25OHD for coating magnetic particles (solid phase) and a vitamin D analogue, 22-carboxy-23,24,25,26,27-pentanorvitamin D3, linked to an isoluminol derivative. This assay is co-specific for 25-hydroxyvitamin D3 and 25-hydroxyvitamin D2. Samples for plasma 25OHD were shipped in three batches to the reference laboratory, with laboratory personnel blinded to case, control or quality control status. For the colorectal cancer case-control study, the inter- and intra-assay coefficient of variations (CVs) were 5.0% and 9.3%, respectively. For the breast cancer case-control subgroup, the inter- and intra-assay CVs were 5.1% and 6.4%, respectively. For the hypertension case-control subgroup, the inter- and intra-assay CVs were 11.2% and 8.1%, respectively.

## 3. Statistical Analysis

### Genetic Variant Selection

Mendelian randomization (genetic variant as an instrumental variable) assumes that the SNP is associated solely with the exposure of interest. In linear regression models, we estimated the association of the five selected 25OHD SNPs and the GRS with BMI and other variables that may influence circulating 25OHD. A five-SNP polygenic additive GRS was created by summing the number (0, 1 or 2) of higher 25OHD-associated alleles (i.e., “high vitamin D alleles”/risk alleles). Therefore, higher values of the GRS were associated with high levels of 25OHD. The unweighted GRS ranged from 1–10. 

We performed Cox proportional-hazard regression models to estimate the association between the individual SNPs and GRS for circulating 25OHD with incident total cancer (*n* = 3985), major site-specific cancers (breast, *n* = 1560; colorectal, *n* = 329; and lung, *n* = 330) and total (*n* = 2973) and cancer (*n* = 770) mortality. We analyzed the GRS continuously, per one-point increase in score, as well as categorically (0–5 (reference group), 6–7 and 8–10 points). The proportionality assumption was verified for each model. Multiple testing was accounted for by using Bonferroni correction, and thus, associations were considered significant if *p* < 0.0008 (0.05/6; 5 SNPs + GRS). Models were adjusted for baseline age (continuous) with an additional model that adjusted for body mass index (BMI). SAS Version 9.3 (SAS Institute, Cary, NC, USA) was used for the analyses. In a sensitivity analysis excluding rs2282679 (GC, vitamin D binding protein) from the GRS, we estimated the hazard ratio (HR) per 20 nmol/L increase in genetically-determined 25OHD to be able to compare results with a previously-published Mendelian randomization evaluating genetically low vitamin D concentrations and mortality [1]. We multiplied the beta and standard error (SE) for association with cancer by the ratio 20/(slope of association with 25OHD). The computer code used is available upon request.

We calculated Mendelian instrumental variable estimates of genetically determined odds ratios by exponentiation of the Wald-type estimator, which is the ratio of the log hazard ratio of the genetic risk score-disease outcome association (incident total cancer and site-specific cancer, total mortality, and cancer mortality) and the linear regression coefficient of the genetic risk score-25OHD association. Standard errors of the Wald-estimator were determined using the delta method. The adjusted 25OHD allele score coefficient came from 1782 participants who had both genotypic and 25OHD measurements. The Mendelian instrumental variable estimate was scaled to 25OHD 20 nmol/L.

## 4. Role of the Funding Sources

The funding sources had no role in the design, conduct or analysis of our study or the decision to submit the manuscript for publication.

## 5. Results

Baseline characteristics are reported in Table 1; all women were of European ancestry with mean (SD) age of 54.7 (7.1) years. 

There were 3985 total (total cancer excluding non-melanoma skin cancer), 1560 breast, 329 colorectal and 330 lung cancer cases that developed, along with 770 cancer deaths. Minor allele frequencies ranged from 16–44% (Table S1: Minor allele frequencies of circulating 25OHD SNPs). Selected SNPs explain 2.6% (*F* = 48, *p* < 0.0001) of the variance in circulating 25OHD between individuals in WGHS. 

### 5.1. Candidate SNP Analyses

Except for the correlation of rs11234027 (DHCR7) with age (*p* = 0.009), we did not identify any significant correlations between genetically-determined 25OHD with age, BMI or physical activity. (Table S2: Concentration of 25OHD according to genotypes used as instrumental variables in GRS (DHCR7/CYP2R1/GC) adjusted for potential confounders individually). However, as expected [1,9], each SNP was associated with circulating 25OHD, and the associations were similar with and without adjustment for vitamin D-associated covariates including BMI (Table S3: Concentration of 25OHD according to genotypes used as instrumental variables in GRS (DHCR7/CYP2R1/GC)). For WGHS participants with 25OHD measures at baseline (*n* = 1782), the concentration of 25OHD by genotype copies of risk allele is reported (Table S4: Concentration of 25-hydroxyvitamin D mean (SD) nmol/L by genotype copies of risk allele in WGHS subgroup (*n* = 1782)). The GRS was associated with circulating 25OHD mean (SD) = 67.8 (26.1) nmol/L, 56.9 (18.7) nmol/L in the lowest quintile versus 73.2 (27.9) nmol/L in the highest quintile of the GRS) (*p* trend < 0.0001 across quintiles). 

None of the five SNPs was associated with incident total, breast, colorectal or lung cancer or total mortality after Bonferroni adjustment for multiple comparisons (Table S5: Cox proportional hazards for incident cancer and mortality by circulating vitamin D SNP per allele associated with increase in 25-hydroxyvitamin D). However, for colorectal cancer, two SNPs, rs12794714 (CYP2R1) and rs10741657 (CYP2R1) had nominally significant associations (HR (95% CI), *p*-values-rs12794714: 1.21 (1.03–1.41), *p* = 0.02 and rs10741657: 1.22 (1.05–1.43), *p* = 0.01). (Table S5: Cox proportional hazards for incident cancer and mortality by circulating vitamin D SNP per allele associated with increase in 25-hydroxyvitamin D) The circulating 25OHD values for the GRS continuous and categorical increased with higher GRS values and categories (Table S6: Mean 25-hydroxyvitamin D level nmol/L for each value of GRS in the case/control cohort (*n* = 1782) and Table S7: Mean 25OHD level nmol/L for each category of GRS in the case/control cohort (*n* = 1782)). The GRS was also not associated with any of the outcomes we evaluated, when analyzed continuously (HR per one unit increase (95% CI), *p* value incident total cancer: 1.01 (1.00–1.03), *p* = 0.17 and total mortality: 1.00 (0.98–1.01), *p* = 0.71) or in categories (Table 2).

The hazard ratios were similar with the addition of BMI to the age-adjusted models for the outcomes we evaluated (Table S8: Cox proportional hazards for cancer and mortality for genetic risk score of alleles associated with increase in circulating 25-hydroxyvitamin D (continuous and categorical) additionally adjusted for BMI). For site-specific cancers, most GRS hazard ratios centered on 1.0 (Table 2). In a sensitivity analysis, we excluded rs2282679 (GC, vitamin D binding protein) from the GRS (range 0–8) and estimated hazard ratios for our targeted outcomes (Table S9: Cox proportional hazards for cancer and mortality for genetic risk score without vitamin D binding protein (GC) alleles associated with increase in circulating 25OHD (continuous and categorical)). Hazard ratios for GRS without GC (Table S9: Cox proportional Hazards for Cancer and Mortality for Genetic Risk Score without vitamin D binding protein (GC) alleles associated with increase in circulating 25OHD (continuous and categorical)) were similar to estimates using GRS with GC (Table 2) with the exception of an increased risk of colorectal cancer for continuous GRS without GC, HR (95% CI) for colorectal cancer (1.08 (1.01–1.15); *p*-value = 0.03) (Table S9: Cox proportional Hazards for Cancer and Mortality for Genetic Risk Score without vitamin D binding protein (GC) alleles associated with increase in circulating 25OHD (continuous and categorical)). We estimated the HR per 20 nmol/L increase in genetically-determined 25OHD to be able to compare results with a previously-published Mendelian randomization evaluating genetically low vitamin D concentrations and mortality in three large Danish cohorts [1]. We found HR (95% CI) for total mortality (0.99 (0.79–1.19)) and cancer mortality (0.99 (0.59–1.39)).

### 5.2. Mendelian Instrumental Variable Estimates

The odds ratio (OR) (95% CI) for a Mendelian genetically determined 20 nmol/L higher plasma 25OHD concentration was 0.97 (0.84–1.13), *p*-value = 0.71, total mortality; 0.98 (0.73–1.32), *p*-value = 0.90, cancer mortality; incident cancer: total, 1.10 (0.96–1.25), *p*-value = 0.17; breast, 1.14 (0.92–1.41), *p*-value = 0.22; colorectal, 1.54 (0.96–2.46), *p*-value = 0.07; lung, 0.96 (0.55–1.68), *p*-value = 0.89. 

## 6. Discussion

We found no significant association between SNPs previously associated with 25OHD and risk of incident total, breast, colorectal and lung cancer or total mortality. There was suggestive evidence for associations of rs12794714 (CYP2R1) and rs10741657 (CYP2R1) with CRC, but these associations did not reach Bonferroni corrected significance. This study had fewer cases than a previous European ancestry Mendelian randomization study [1] that showed an inverse relationship between these vitamin D SNPs and total and cancer mortality. In a sensitivity analysis, we looked at the same allele score, by excluding rs2282679 (GC, vitamin D binding protein), that Afzai et al. [1] evaluated in a Danish population of 95,766 European participants with 10,349 deaths. We estimated the Mendelian OR per 20 nmol/L increase in genetically-determined 25OHD, and the OR (95% CI) for total mortality (0.97 (0.84–1.13)) and cancer mortality (0.98 (0.73–1.32)) were statistically different from the point estimates of the Afzai study [1] (OR (95% CI) per 20 nmol/L lower plasma 25OHD concentration; total mortality: 1.30 (1.05–1.61) and cancer mortality 1.43 (1.02–1.99)). Afzai et al.’s findings correspond to a 30% reduced cancer mortality for a genetically 20 nmol/L increase in 25OHD concentration. 

The genetic increment may be modified by the underlying vitamin D status of the population (e.g., limited sun exposure and limited vitamin D in diet); the underlying vitamin D status of the U.S. population may differ from that of the Copenhagen population. Thus, the incremental change in circulating vitamin D by GRS may have different implications for different populations based on their average vitamin D levels. A pooled analysis (2304 participants) of a randomized trial and prospective cohort study reported that women with 25OHD concentrations >100 nmol/L had a 67% lower risk of all invasive cancers combined (excluding skin cancer) than women with concentrations <50 nmol/L [16]. Similarly, a meta-analysis of studies evaluating circulating vitamin D levels, functionally relevant vitamin D receptor genetic variants and variants within vitamin D pathway genes and cancer survival or disease progression showed a benefit of higher vitamin D levels on survival [17]. In contrast, Skaaby et al. prospectively reported no association between vitamin D status and incident total and specific type of cancer [18] or mortality [19] in three cohorts from the general Danish population. The Vitamin D and Omega-3 Trial (VITAL) [20] is expecting a 50 nmol/L increase in 25OHD with vitamin D supplementation, 2000 IU/day, and should be sufficiently powered to detect reduced cancer mortality if the Afzai results are applicable to a randomized vitamin D supplementation clinical trial. Yet, recent meta-analyses of randomized vitamin D intervention trials found little effect of vitamin D supplementation on mortality endpoints [2,3].

Mendelian randomization analyses of genetic variants, which likely reflect lifetime, biologic exposures, represent a complementary approach to testing vitamin D-cancer hypotheses through trials of vitamin D supplementation. Our study agrees with prior studies that suggested that circulating vitamin D genetic markers account for only 2–5% of the variance in 25OHD [4,5,21]. Since the effect sizes of individual alleles are often small, the predictive value of a single variant of a small effect on circulating 25OHD levels is negligible [22]. In one study, four (rs11234027, rs12794714, rs7944926, rs12794714) of the five SNPS used in our study explained more variation in circulating 25OHD (5.2%) than a polygenic score including 9000 SNPs (0.16%) [9]. Our GRS instrumental variable *F* statistic = 40, which is generally considered a strong genetic instrument measurement [23]. Yet, some biostatisticians suggest that even with an *F* statistic >10, the results from Mendelian randomization may lead to wrong inferences [23].

Although family studies have estimated the heritability of circulating 25OHD ranging from 43–80% [24,25,26,27], the known SNPs account for only about 5% of the variance in 25OHD and highlight the complex trait of circulating 25OHD. The majority of the genetic effect for circulating 25OHD may be related to rare variants, structural variants other than SNPs or gene-environment interactions [28,29]. It is also possible that other genetic variants affect circulating 25OHD and cancer risk through entirely different mechanisms independent of 25OHD levels. We assumed an additive allelic effect and do not account for potential gene-gene interactions in our Cox proportional models because we did not see a main association of GRS with cancer and mortality.

Our findings do not support an association between vitamin D status, as reflected by 25OHD-related genotypes, and breast cancer risk. Our findings are in agreement with the Women’s Health Initiative (WHI) vitamin D and calcium intervention trial, which reported no effect of vitamin D and calcium supplementation on incident invasive postmenopausal breast cancer [30]. Limitations of the WHI trial included compliance, the low vitamin D dose used and duration of the trial (average of seven years) [31]. Our study includes a longer period of follow-up than prior studies and is embedded within a chemoprevention randomized clinical trial of aspirin and vitamin E. Further, the vitamin D genetic score was unrelated to breast cancer risk based on 9500 cases and 11,000 controls in a multicohort analysis that included WGHS participants [32]. Our study is a prospective analysis compared to this retrospective case-control multicohort analysis. In case-control analyses, one cannot predict whether exposure to the risk factor, circulating 25OHD, preceded development of the cancer. We may not have observed an association of our GRS with breast cancer because of the weak association of plasma 25OHD with breast cancer, resulting in an underpowered analysis. In a meta-analysis of prospective studies including nested case-control studies and cohort studies, every 25 nmol/L increase in serum 25OHD concentration significantly reduced breast cancer risk by 3.2% [33].

Our null findings for colorectal cancer are consistent with a pooled analysis of 13 studies (WGHS participants not included) included in the Genetics and Epidemiology of Colorectal Cancer Consortium (GECCO) and Colon Cancer Family Registry (CCFR) with about 10,000 cases and 13,000 controls that demonstrated no association between genetic markers of circulating 25OHD and colorectal cancer [21]. Furthermore, randomized clinical trials of vitamin D supplementation, including the Women’s Health Initiative [34] and a British trial [35] showed no effect on total or colorectal cancer incidence or total mortality. In contrast, cohort studies have generally shown an inverse association between high circulating 25OHD and colorectal cancer risk [36]. The Circulating Biomarkers and Breast and Colorectal Cancer Consortium (BBC3), a large international pooling project of 21 cohorts with absolute concentrations of circulating vitamin D measured in prediagnostic samples in approximately 10,000 breast cancer and 6000 colorectal cancer cases and their matched controls, and the VITAL study [20] may offer additional and more conclusive insights for colorectal and breast cancer. 

### 6.1. Strengths

The strengths of our study are the prospective design and homogeneous nature of the WGHS cohort. Our genetic instrumental variable was robustly associated with circulating 25OHD. Genotypes, randomly distributed at birth, are unlikely to be confounded by lifestyle or environmental factors such as poor nutrition or inactivity, which is a noted strength of the Mendelian randomization approach. 

### 6.2. Limitations

Statistically-significant effect estimates for specific cancer sites are difficult to establish unless large magnitudes of association are observed, or large sample sizes are achieved. While we had a large sample size, the HRs close to one suggest there was no overall effect for the vitamin D GRS on cancer endpoints in WGHS. Yet, this study adds to the current literature, because WGHS total mortality and cancer mortality data have not been included in previous vitamin D genetic meta-analyses [16]. A limitation of the current analysis is that we do not evaluate the structural and functional impact of these five SNPs through in silico models that examine coding variants associated with change in protein function and activity. 

## 7. Conclusions

A genetic risk score for higher 25OHD blood levels was not associated with cancer incidence or mortality in this large cohort of U.S. women. Our findings do not provide support for a causal association between vitamin D status, as reflected by 25OHD-related genotypes, and cancer risk. Future research is needed to investigate the efficacy of vitamin D supplementation in reducing cancer risk, as well as the role of vitamin D-related genetic variation in the setting of vitamin D supplementation. VITAL [20] and other ongoing large-scale randomized trials of vitamin D supplementation have the potential to address these important questions, as well as to elucidate some of the relevant biologic mechanisms.

## Figures and Tables

**Table 1:** Baseline characteristics of Women’s Genome Health Study European ancestry participants (*n* = 23,294) *.

Age (Years)	54.7 (7.1)
Randomized aspirin, %	50
Randomized vitamin E, %	50
Season of blood draw, %	
Winter	32.1
Spring or fall	17.2
Summer	50.7
HRT ^1^ use, never, %	48.3
No oral contraceptive use, %	30.1
Postmenopausal, %	54.5
Body mass index (kg/m^2^)	25.9 (5.0)
Exercise (METS-h/week) ^2^	14.2 (18.3)
Alcohol (g/day)	4.3 (8.4)
Total vitamin D intake (IU/day)	354.7 (242.8)
Vitamin D without supplement (IU/day)	236.1 (111.2)
Smoking, %	
Current	11.6
Past	37.5
Never	50.9
Family history of colorectal cancer ^3^, %	10.6
Family history of breast cancer, %	6.3
Mammogram screening, %	62.7
Colonoscopy or sigmoidoscopy screening ^4^, %	8.0
Diabetes, %	2.5

* Values represent the mean and standard deviation (SD) unless otherwise specified; ^1^ hormone replacement therapy (HRT) use at baseline; ^2^ metabolic equivalents (METS)-hours per week; ^3^ family history of colorectal cancer in first degree relative at baseline; ^4^ history of colonoscopy or sigmoidoscopy screening for screening or symptoms at baseline.

**Table 2:** Cox proportional hazards for cancer and mortality for genetic risk score of alleles associated with an increase in circulating 25OHD (continuous and categorical) *.

	Cases/Sample Size	Rate/1000 pyrs ^1^	HR (95% CI)
Breast			
Continuous	1560/23,294		1.02 (0.99–1.05)
Reference	417/6477	3.56	1.00
GRS 6–7	626/9619	3.60	1.02 (0.90–1.15)
GRS 8–10	517/7196	4.00	1.13 (0.99–1.28)
Colorectal			
Continuous	329/23,294		1.06 (1.00–1.13)
Reference	83/6477	0.69	1.00
GRS 6–7	136/9621	0.76	1.12 (0.85–1.47)
GRS 8–10	110/7195	0.82	1.21 (0.91–1.61)
Lung			
Continuous	330/23,294		1.00 (0.94–1.06)
Reference	99/6477	0.82	1.00
GRS 6–7	129/9621	0.72	0.89 (0.68–1.15)
GRS 8–10	102/7196	0.76	0.94 (0.71–1.23)
Total			
Continuous	3985/23,294		1.01 (1.00–1.03)
Reference	1091/6468	9.71	1.00
GRS 6–7	1626/9610	9.76	1.01 (0.93–1.09)
GRS 8–10	1268/7183	10.23	1.06 (0.98–1.15)
Total Mortality			
Continuous	2973/23,294		1.00 (0.98–1.02)
Reference	850/6477	6.87	1.00
GRS 6–7	1193/9621	6.49	0.96 (0.88–1.05)
GRS 8–10	930/7196	6.77	1.00 (0.91–1.09)
Cancer mortality			
Continuous	770/23,294		1.00 (0.96–1.04)
Reference	217/6468	1.93	1.00
GRS 6–7	305/9610	1.83	0.95 (0.80–1.13)
GRS 8–10	248/7183	2.00	1.05 (0.87–1.26)

* Adjusted for age; Reference is genetic risk score (GRS) = 0–5. ^1^ Person-years (pyrs).

## Supplementary Materials

The following are available online at www.mdpi.com/2072-6643/10/1/55/s1, Table S1: Minor allele frequencies of circulating 25OHD SNPs, Table S2: Concentration of 25OHD according to genotypes used as instrumental variables in GRS (DHCR7/CYP2R1/GC) adjusted for potential confounders individually Table S3: Concentration of 25OHD according to genotypes used as instrumental variables in GRS (DHCR7/CYP2R1/GC), Table S4: Concentration of 25-hydrovyvitamin D mean (SD) nmol/L by genotype copies of risk allele in WGHS subgroup (*n* = 1782), Table S5: Cox proportional hazards for incident cancer and mortality by circulating vitamin D SNP per allele associated with increase in 25-hydroxyvitamin D, Table S6: Mean 25-hydroxyvitamin D level nmol/L for each value of GRS in the case/control cohort (*n* = 1782), Table S7: Mean 25OHD level nmol/L for each category of GRS in the case/control cohort (*n* = 1782), Table S8: Cox proportional hazards for cancer and mortality for Genetic Risk Score of alleles associated with increase in circulating 25-hydroxyvitamin D (continuous and categorical) additionally adjusted for BMI, Table S9: Cox proportional Hazards for Cancer and Mortality for Genetic Risk Score without vitamin D binding protein (GC) alleles associated with increase in circulating 25OHD (continuous and categorical).
