# Supplementary material for: Association between Vitamin D Genetic Risk Score and Cancer Risk in a Large Cohort of U.S. Women
Source: Nutrients. 2018 Jan 9;10(1):55. doi: 10.3390/nu10010055 (PMC5793283; doi:10.3390/nu10010055)
Supplement: Supplementary file 1 [file nutrients-10-00055-s001.pdf]

Supplemental Tables

Table S1. Minor allele frequencies of circulating 25OHD SNPs.

SNP	Position (nearest gene)	Major/minor allele	Minor allele frequency mean [a]
rs11234027	DHCR7	G/A	0.16
rs7944926	DHCR7	G/A	0.25
rs12794714	CYP2R1	G/A	0.44
rs10741657	CYP2R1	A/G	0.39
rs2282679	GC	T/G	0.28

[a] Allele associated with decreasing circulating 25OHD.

Table S2. Pearson correlation coefficients [a].

SNP	Position (nearest gene)	Age,years	BMI. Kg/m^2	Exercise, METs/wk
rs11234027	DHCR7	-0.06[b]	0.02	-0.02
rs7944926	DHCR7	0.01	-0.03	-0.01
rs12794714	CYP2R1	0.01	0.03	-0.04
rs10741657	CYP2R1	-0.02	-0.02	0.05
rs2282679	GC	0.01	0.002	0.02

[a] all p values >0.05 for Pearson correlation unless otherwise noted; [b] p=0.009.

Table S3. Concentration of 25OHD according to genotypes used as instrumental variables in GRS (DHCR7/CYP2R1/GC).

SNP		Beta(SE) Without BMI	P value	Beta(SE) With BMI	P value
rs11234027	DHCR7	4.1(1.2)	0.0004	4.3(1.1)	0.0002
rs7944926	DHCR7	-3.5(0.96)	0.0003	-3.7(0.94)	<0.0001
rs12794714	CYP2R1	3.3(0.85)	0.0001	3.5(0.82)	<0.0001
rs10741657	CYP2R1	-3.9(0.85)	<0.0001	-4.0(0.82)	<0.0001
rs2282679	GC	6.0(0.91)	<0.0001	6.1(0.88)	<0.0001

Adjusted for age, year of birth, season, case/control status. SNPs are coded on major allele, referent for season is winter, referent for case-control status is pilot study with and without BMI in regression model. N=1782 European ancestry with successful genotyping.

Table S4. Concentration of 25-hydrovyvitamin D mean (SD) nmol/L by genotype copies of risk allele in WGHS subgroup (N=1,782).

SNP	Position (nearest gene)	N	Mean(SD)
rs11234027	DHCR7		
0		32	60.5(28.5)
1		502	65.1(25.5)
2		1248	69.0(26.2)
rs7944926	DHCR7		
0		992	69.4(26.4)
1		676	66.4(25.5)
2		114	62.0(26.0)
rs10741657	CYP2R1		
0		284	71.4(28.6)
1		832	68.6(27.3)
2		666	65.2(23.0)
rs12794714	CYP2R1		
0		335	63.8(21.7)
1		879	68.3(26.1)
2		568	69.4(28.1)
rs2282679	GC		
0		161	59.9(21.7)
1		737	65.1(25.7)
2		884	71.4(26.6)

Table S5. Cox proportional hazards for incident cancer and mortality by circulating vitamin D SNP per allele associated with increase in 25-hydroxyvitamin D.

SNP	Position (nearest gene)	Beta(SE)	HR(95%CI)	P value
Incident Cancer				
Breast				
rs11234027	DHCR7	0.083(0.051)	1.09(0.98-1.20)	0.10
rs7944926	DHCR7	0.051(0.042)	1.05(0.97-1.14)	0.22
rs12794714	CYP2R1	0.021(0.036)	1.02(0.95-1.10)	0.55
rs10741657	CYP2R1	0.002(0.037)	1.00(0.93-1.08)	0.96
rs2282679	GC	0.011(0.040)	1.01(0.93-1.09)	0.79
Colorectal				
rs11234027	DHCR7	-0.016(0.106)	0.98(0.80-1.21)	0.88
rs7944926	DHCR7	0.065(0.091)	1.07(0.89-1.28)	0.47
rs12794714	CYP2R1	0.188(0.079)	1.21(1.03-1.41)	0.02
rs10741657	CYP2R1	0.200(0.078)	1.22(1.05-1.43)	0.01
rs2282679	GC	-0.072(0.086)	0.93(0.79-1.10)	0.41
Lung				
rs11234027	DHCR7	0.034(0.108)	1.04(0.84-1.28)	0.75
rs7944926	DHCR7	0.058(0.090)	1.06(0.89-1.27)	0.52
rs12794714	CYP2R1	-0.041(0.078)	0.96(0.82-1.12)	0.59
rs10741657	CYP2R1	-0.017(0.080)	0.98(0.84-1.15)	0.83
rs2282679	GC	-0.039(0.086)	0.96(0.81-1.14)	0.65
Total				
rs11234027	DHCR7	0.032(0.031)	1.03(0.97-1.10)	0.30
rs7944926	DHCR7	0.033(0.026)	1.03(0.98-1.09)	0.20
rs12794714	CYP2R1	0.023(0.022)	1.02(0.98-1.07)	0.30
rs10741657	CYP2R1	0.029(0.023)	1.03(0.98-1.08)	0.21
rs2282679	GC	-0.019(0.025)	0.98(0.94-1.03)	0.46
Mortality				
Total mortality				
rs11234027	DHCR7	0.025(0.036)	1.03(0.96-1.10)	0.48
rs7944926	DHCR7	0.019(0.030)	1.02(0.96-1.08)	0.53
rs12794714	CYP2R1	-0.030(0.026)	0.97(0.92-1.02)	0.24
rs10741657	CYP2R1	-0.004(0.027)	1.00(0.95-1.05)	0.88
rs2282679	GC	-0.022(0.029)	0.98(0.92-1.04)	0.44
Cancer mortality				
rs11234027	DHCR7	0.107(0.072)	1.11(0.97-1.28)	0.14
rs7944926	DHCR7	0.013(0.059)	1.01(0.90-1.14)	0.82
rs12794714	CYP2R1	-0.067(0.051)	0.93(0.85-1.03)	0.18
rs10741657	CYP2R1	-0.004(0.052)	1.00(0.90-1.10)	0.94
rs2282679	GC	-0.012(0.057)	0.99(0.88-1.11)	0.83

All SNPs are coded on the allele in the direction of increasing 25OHD per direction of the beta from linear regressions of 25OHD regressed on each of the 5 SNPs (age adjusted). HR calculated in reference to the allele associated with increased 25OHD.

Table S6. Mean 25-hydroxyvitamin D level nmol/L for each value of GRS in the case/control cohort (N=1782).

GRS	N	Mean(SD)	Minimum/Maximum 25OHD nmol/L
0-1	3	63.1(16.0)	47.2/79.2
2	26	56.2(19.1)	19.7/107.6
3	64	58.3(22.2)	14.2/124.8
4	155	62.1(21.9)	18.2/159.4
5	260	65.4(24.2)	12.0/150.0
6	362	65.0(23.4)	17.0/169.5
7	366	69.8(28.7)	10.2/221.9
8	312	72.3(27.5)	19.7/178.5
9	159	71.2(26.8)	20.7/171.5
10	75	77.3(29.9)	26.2/181.7

Table S7. Mean 25OHD level nmol/L for each category of GRS in the case/control cohort (N=1782).

GRS	N	Mean(SD)	Minimum/Maximum 25OHD nmol/L
0-5	508	63.0(23.1)	12.0/159.4
6-7	728	67.4(26.2)	10.2/221.9
8-10	546	72.7(27.6)	19.7/181.7

Table S8. Cox proportional hazards for cancer and mortality for Genetic Risk Score of alleles associated with increase in circulating 25-hydroxyvitamin D (continuous and categorical) additionally adjusted for BMI.

	Cases/sample size	Rate/1000pyrs	HR (95% CI)
Breast			
Continuous	1560/23,294		1.02(0.99-1.05)
Reference	417/6477	3.56	1.00
GRS 6-7	626/9619	3.60	1.02(0.90-1.15)
GRS 8-10	517/7196	4.00	1.13(0.99-1.28)
Colorectal			
Continuous	329/23,294		1.06(1.00-1.13)
Reference	83/6477	6.90	1.00
GRS 6-7	136/9621	7.62	1.11(0.85-1.46)
GRS 8-10	110/7195	8.24	1.20(0.91-1.60)
Lung			
Continuous	330/23,294		1.00(0.94-1.06)
Reference	99/6477	0.82	1.00
GRS 6-7	129/9621	0.72	0.90(0.69-1.17)
GRS 8-10	102/7196	0.76	0.95(0.72-1.25)
Total			
Continuous	3985/23,294		1.01(1.00-1.03)
Reference	1091/6468	9.71	1.00
GRS 6-7	1626/9610	9.76	1.01(0.93-1.09)
GRS 8-10	1268/7183	10.23	1.06(0.98-1.15)
Total Mortality			
Continuous	2973/23,294		1.00(0.98-1.02)
Reference	850/6477	6.87	1.00
GRS 6-7	1193/9621	6.49	0.96(0.88-1.05)
GRS 8-10	930/7196	6.77	0.99(0.91-1.09)
Cancer mortality			
Continuous	770/23,294		1.00(0.96-1.04)
Reference	217/6468	1.93	1.00
GRS 6-7	305/9610	1.83	0.96(0.80-1.14)
GRS 8-10	248/7183	2.00	1.05(0.88-1.26)
Adjusted for age and BMI			

Table S9. Cox proportional Hazards for Cancer and Mortality for Genetic Risk Score without vitamin D binding protein (GC) alleles associated with increase in circulating 25OHD (continuous and categorical).

	Cases/sample size	Rate/1000pyrs	HR (95% CI)
Breast			
Continuous	1560/23,294		1.02(0.99-1.05)
Reference	247/3608	3.78	1.00
GRS 4-5	595/9342	3.53	0.94(0.81-1.09)
GRS 6-8	718/10,342	3.85	1.02(0.89-1.18)
Colorectal			
Continuous	329/23,294		1.08(1.01-1.15)
Reference	42/3608	6.25	1.00
GRS 4-5	131/9344	7.57	1.24(0.87-1.75)
GRS 6-8	156/10,341	8.13	1.31(0.93-1.85)
Lung			
Continuous	330/23,294		1.00(0.94-1.07)
Reference	57/3608	0.85	1.00
GRS 4-5	130/9344	0.75	0.90(0.66-1.23)
GRS 6-8	102/7196	0.74	0.88(0.65-1.20)
Total			
Continuous	3985/23,294		1.02(1.00-1.04)
Reference	613/3601	9.77	1.00
GRS 4-5	1567/9335	9.69	1.00(0.91-1.10)
GRS 6-8	1805/10325	10.12	1.04(0.95-1.14)
Total Mortality			
Continuous	2973/23,294		1.00(0.98-1.02)
Reference	483/3608	6.99	1.00
GRS 4-5	1153/9344	6.47	0.96(0.87-1.07)
GRS 6-8	1337/10,342	6.77	0.98(0.88-1.09)
Cancer mortality			
Continuous	770/23,294		1.00(0.96-1.04)
Reference	126/3601	2.01	1.00
GRS 4-5	298/9335	1.84	0.92(0.75-1.13)
GRS 6-8	346/10.325	1.94	0.97(0.79-1.19)
